# Pterostilbene attenuates lung ischemia-reperfusion injury: integrative insights from network pharmacology, molecular dynamics, and experimental validation

**DOI:** 10.3389/fphar.2026.1747977

**Published:** 2026-04-01

**Authors:** Haotian Bai, Heng Zhao, Jinteng Feng, Hongyi Wang, Yixing Li, Zhe Chen, Bin He, Chi Wang, Rui Gao, Rui Zhao, Shan Gao, Guangjian Zhang

**Affiliations:** 1 Department of Thoracic Surgery, The First Affiliated Hospital of Xi’an Jiaotong University, Xi’an, China; 2 Key Laboratory of Enhanced Recovery After Surgery of Integrated Chinese and Western Medicine, Administration of Traditional Chinese Medicine of Shaanxi Province, The First Affiliated Hospital of Xi’an Jiaotong University, Xi’an, China; 3 Department of Nuclear Medicine, The First Affiliated Hospital of Xi’an Jiaotong University, Xi’an, China

**Keywords:** pterostilbene, lung ischemia-reperfusion injury, network pharmacology, molecular dynamics simulations, experimental validation

## Abstract

**Background:**

Lung ischemia-reperfusion injury (LIRI) is a principal cause of primary graft dysfunction (PGD) following lung transplantation, severely compromising recipient survival. However, effective therapies remain unavailable due to its complex pathophysiology. Pterostilbene (PTE) is a natural stilbene compound known for its well-documented anti-inflammatory, antioxidant, and antitumor properties. However, its effects and underlying mechanisms in LIRI remain unclear.

**Methods:**

Potential targets of PTE and LIRI were retrieved from multiple public databases, followed by network analysis and functional enrichment to identify core targets and pathways. Molecular docking and dynamics simulations were conducted to assess the binding affinity and stability between PTE and its core targets. Finally, a rat left hilar clamping model and an OGD/R model in BEAS-2B cells were employed to experimentally validate the protective effects and molecular mechanisms of PTE.

**Results:**

A total of 104 intersecting targets were identified with ten core genes such as PIK3CB and MAPK8 highlighted. Gene Ontology and KEGG analyses revealed significant enrichment in apoptosis- and inflammation-related pathways, particularly PI3K/AKT and MAPK signaling. Docking and simulation results demonstrated stable binding of Pterostilbene to core targets (binding energy ≤−5.6 kcal/mol). *In vivo*, PTE alleviated IR-induced lung injury, reduced pulmonary edema, apoptosis, and pro-inflammatory cytokine release. *In vitro*, PTE enhanced cell viability, decreased the levels of pro-inflammatory cytokines, inhibited Caspase-3 activation and Bax expression, and increased Bcl-2 levels. Mechanistically, PTE promoted PI3K/AKT activation while suppressing JNK/c-Jun phosphorylation both *in vivo* and *in vitro*. Notably, LY294002 (a PI3K inhibitor) and Anisomycin (a JNK activator) partially reversed the anti-apoptotic and anti-inflammatory effects of PTE, respectively.

**Conclusion:**

This study provides the first integrated evidence combining network pharmacology and experimental validation that PTE protects against LIRI by modulating the PI3K/AKT and JNK/c-Jun signaling pathways, offering novel pharmacological insights into its translational potential in LIRI.

## 1 Introduction

Lung transplantation (LTx) remains the most effective therapeutic option for patients with end-stage lung diseases (Christie et al., 2024). However, primary graft dysfunction (PGD), a common and severe early postoperative complication, substantially compromises survival outcomes in LTx recipients compared with other solid organ transplant recipients (Chacon-Alberty et al., 2023; Valapour et al., 2025). Lung ischemia-reperfusion injury (LIRI), occurring upon restoration of blood flow following ischemia, is widely regarded as the primary initiating event of PGD (Vlastos et al., 2022; Zhao et al., 2022). The pathophysiology of LIRI remains incompletely understood but is thought to involve oxidative stress, calcium overload, endoplasmic reticulum stress, coagulation dysfunction, and excessive immune activation, ultimately leading to various forms of cell death and impaired graft function (Chen-Yoshikawa, 2021; de Perrot et al., 2003; den Hengst et al., 2010). Currently, no effective pharmacological interventions are available, underscoring the urgent need to explore novel therapeutic strategies.

Natural plant compounds have garnered considerable attention due to their efficacy and favorable safety profiles in various diseases, offering promising approaches for ischemia-reperfusion injury. Pterostilbene (3,5-dimethoxy-4-hydroxystilbene, PTE), a naturally dimethylated analog of resveratrol, is primarily found in blueberries and also present in grapes and peanuts (Lin et al., 2020). Compared with resveratrol, PTE exhibits superior bioactivity, bioavailability, and metabolic stability due to the substitution of two hydroxyl groups with methoxy groups in the structure (Ali et al., 2025; Huang et al., 2025; Nagarajan et al., 2022), enhancing its therapeutic potential. Accumulating evidence demonstrates that PTE possesses multiple pharmacological properties, including anti-inflammatory, antioxidant, antitumor, anti-aging effects, and regulation of glucose and lipid metabolism, supporting its benefits in cancer, diabetes, cardiovascular, and neurological diseases (Chen et al., 2024; Dou et al., 2024; Dutta et al., 2023; He et al., 2024; Kang et al., 2025; Shen et al., 2023; Xi et al., 2025; Zhang et al., 2023). Notably, recent studies (Cheng et al., 2016; Wu et al., 2017; Yan et al., 2021; Yang et al., 2023; Yu et al., 2017) have also demonstrated that PTE offer protective benefits against IR injury through multiple mechanisms, particularly in the brain and myocardium, including inhibition of oxidative stress and inflammation, attenuation of apoptosis, preservation of endothelial cytoskeleton and basement membrane integrity, and improvement of mitochondrial function. However, the application of PTE in LIRI models remains underexplored, with current research on its role in pulmonary diseases largely focusing on lung cancer and acute lung injury induced by virus or lipopolysaccharide (Kang et al., 2025; Zhang et al., 2020; Zhang et al., 2024).

Network pharmacology integrates cheminformatics, bioinformatics, systems biology, and pharmacology to systematically identify potential targets and pathways of complex natural compounds, providing a solid theoretical foundation for subsequent *in vitro* and *in vivo* validation (Hopkins, 2007; Zhang et al., 2017). As a single compound with a well-defined molecular structure, PTE eliminates the complexities arising from multi-component interference, thereby enhancing the precision and efficiency of network pharmacology analysis. Furthermore, molecular docking (Paggi et al., 2024; Sivakumar et al., 2020) and molecular dynamics (MD) simulations (Hollingsworth and Dror, 2018; Moradi et al., 2024; Wu et al., 2022) can offer theoretical support for drug-target interactions through evaluating binding affinity and conformational stability. The integration of these approaches allows a systematic elucidation of the underlying molecular mechanisms and therapeutic potential.

In this study, we applied an integrated strategy combining network pharmacology, molecular docking, and MD simulations to identify core targets and signaling pathways of PTE in LIRI. The predicted mechanisms were further validated using a rat left hilar clamping model and an oxygen-glucose deprivation/reoxygenation (OGD/R) model in BEAS-2B cells. Our findings indicate that the protective effects of PTE are primarily mediated through anti-apoptotic and anti-inflammatory actions, potentially via activation of the PI3K/AKT pathway and inhibition of the JNK/c-Jun pathway. In summary, this study systematically demonstrates, for the first time, the protective role of PTE against LIRI and its molecular mechanisms, providing a novel theoretical basis for the therapeutic potential of natural compounds in LIRI.

## 2 Results

### 2.1 Acquisition of PTE and LIRI targets, PPI network construction, and core targets identification

**FIGURE 1:**
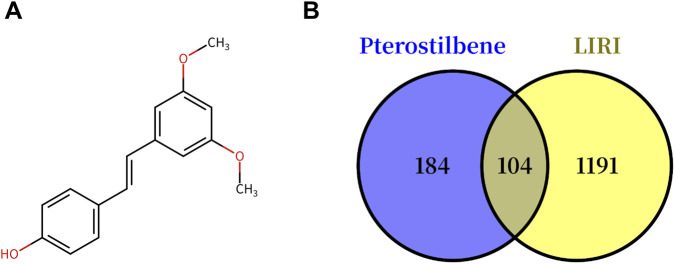
Chemical structure of PTE and Venn analysis. **(A)** The 2D chemical structure of Pterostilbene (C_16_H_16_O_3_), CAS number: 537-42-8. **(B)** Venn diagram representing the overlapping of PTE targets (blue) and LIRI targets (yellow). PTE: pterostilbene, 2D: two-dimensional, CAS: chemical abstracts service.

The molecular formula of PTE is C_16_H_16_O_3_, and its structure is shown in Figure 1A. Potential targets of PTE were retrieved from PharmMapper, SwissTargetPrediction, and TCMSP, yielding a total of 289 unique targets. For LIRI, 1,295 disease-related targets were obtained from OMIM, GeneCards, and TTD after removing duplicates. A total of 104 intersecting targets between PTE and LIRI were identified via Venny analysis (Figure 1B; Supplementary Table S2), representing the potential targets of PTE in treating LIRI.

**FIGURE 2:**
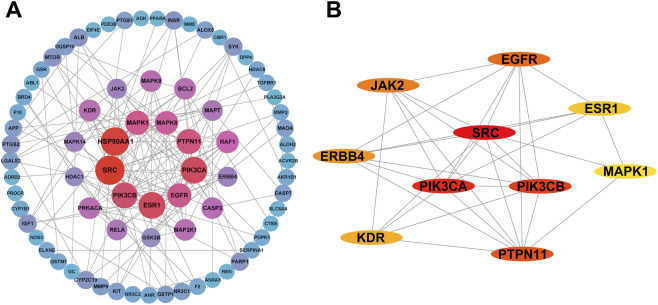
PPI network analysis and identification of core targets. **(A)** The PPI network was constructed based on the STRING database and visualized in Cytoscape software. **(B)** The top 10 core targets were identified from the PPI network using the Network Analyzer plugin. Each circular node represents a protein, and the connecting lines indicate protein-protein interactions. Larger nodes reflect higher degree values, whereas darker colors denote stronger interactions with other targets. PPI: protein-protein interaction.

To assess the priority of common targets, all 104 were imported into the STRING database for protein-protein interaction (PPI) network analysis with a confidence score ≥0.90. The resulting network comprised 104 nodes and 166 edges, with an average node degree of 3.19 and a PPI enrichment *P*-value < 1.0 × 10^−16^, indicating significant interaction enrichment (Supplementary Figure S2; Supplementary Table S3). In the network, nodes represent proteins and edges represent interactions, with the number of edges reflecting the degree of correlation. Core targets were further identified using the CytoNCA plugin in Cytoscape 3.10.2 software based on degree, betweenness, and closeness centrality (Figure 2A; Supplementary Table S4). The Network Analyzer plugin was also applied for supplementary validation (Figure 2B; Supplementary Table S5), while subsequent experiments were conducted based on the CytoNCA plugin to ensure result consistency. Based on network topology analysis (Supplementary Table S4) and the biological relevance of the targets to LIRI, particularly their involvement in pathways such as oxidative stress, inflammation, and cell death, the top 10 potential core targets (SRC, HSP90AA1, PIK3CA, ESR1, PIK3CB, PTPN11, EGFR, MAPK1, MAPK8, and RAF1) were selected for further investigation.

### 2.2 Functional enrichment analysis of potential targets for PTE treatment in LIRI

**FIGURE 3:**
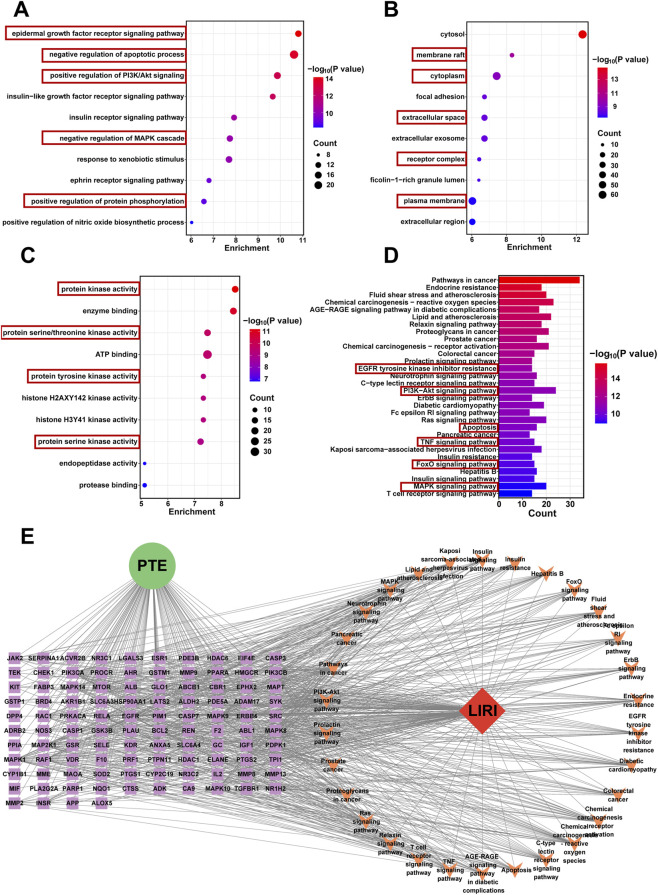
GO and KEGG enrichment analyses and CTPD network construction. **(A–C)** Bubble plots of GO enrichment analysis showing biological process (BP), cellular component (CC), and molecular function (MF), respectively. **(D)** KEGG pathway enrichment analysis. **(E)** The CTPD network was constructed in Cytoscape based on intersecting targets and KEGG-enriched pathways. In the network, green circles represent the compound (PTE), red diamonds denote the disease (LIRI), purple rectangles indicate targets, and orange arrows represent pathways. Edges between nodes illustrate their interactions. GO: Gene Ontology, KEGG: Kyoto Encyclopedia of Genes and Genomes, CTPD: compound-target-pathway-disease.

To explore the potential pharmacological mechanisms of PTE against lung ischemia-reperfusion injury, the 104 intersecting targets were subjected to Gene Ontology (GO) and Kyoto Encyclopedia of Genes and Genomes (KEGG) enrichment analyses using the DAVID database. Under the criteria of *P* < 0.05 and *Q* < 0.2, GO analysis identified 470 biological process (BP) terms, 69 cellular component (CC) terms, and 161 molecular function (MF) terms. The top 10 enriched terms for each category were shown in Figures 3A–C. In the BP category, key processes included epidermal growth factor receptor signaling pathway, negative regulation of apoptotic process, positive regulation of PI3K/AKT signaling, negative regulation of MAPK cascade, and positive regulation of protein phosphorylation (Figure 3A). CC analysis revealed significant enrichment in membrane rafts, cytoplasm, extracellular space, receptor complexes, and plasma membrane (Figure 3B). In MF analysis, protein serine/threonine kinase activity and protein tyrosine kinase activity were among the most enriched functions (Figure 3C).

Furthermore, KEGG pathway enrichment identified 160 signaling pathways potentially involved in PTE-mediated protection against LIRI, with the top 30 pathways illustrated in Figure 3D. Interestingly, these enriched pathways are mainly associated with cell death (such as Apoptosis, EGFR tyrosine kinase inhibitor resistance, PI3K-AKT signaling pathway and FoxO signaling pathway) and inflammation (such as TNF signaling pathway and MAPK signaling pathway), which are the two most prominent signaling alterations during LIRI (Capuzzimati et al., 2022). Indeed, the subsequent examination (Figure 8) of the PI3K/AKT and MAPK pathways further supported the reliability of the enrichment analysis. In addition, based on the key targets and enriched pathways, a compound-target-pathway-disease (CTPD) network was constructed in Cytoscape to visualize the interactions among PTE, targets, pathways, and LIRI (Figure 3E). In summary, these analyses suggest that PTE may mitigate LIRI by modulating apoptosis and inflammation related pathways, offering both potential directions and a theoretical foundation for investigating its protective mechanisms.

### 2.3 Molecular docking and molecular dynamics simulations of PTE with key targets

**FIGURE 4:**
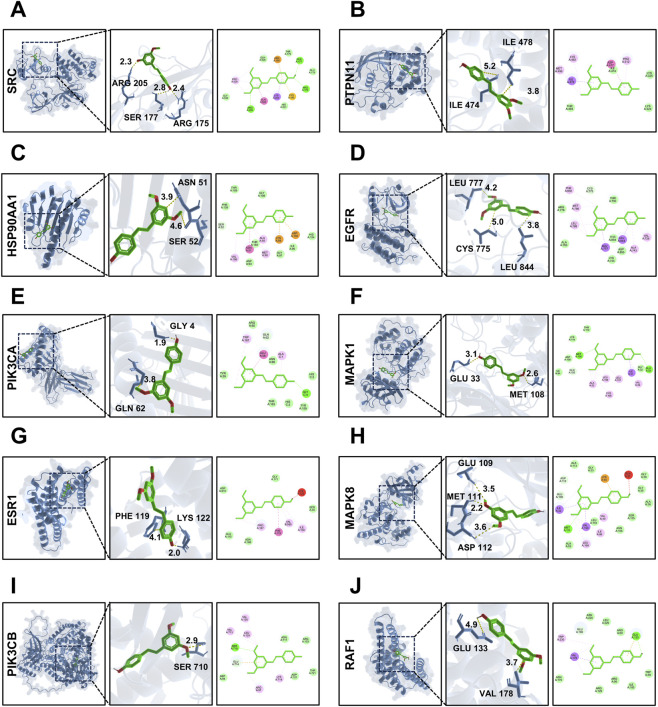
Molecular docking results of PTE with the top 10 core targets. **(A)** PTE -SRC (PDB: 1FMK), BE: −6.7 **(B)** PTE-PTPN11 (PDB: 3ZM1), BE: −5.8. **(C)** PTE- HSP90AA1 (PDB: 5J80), BE: −6.1. **(D)** PTE-EGFR (PDB: 8A27), BE: −7.4. **(E)** PTE-PIK3CA (PDB: 9ASF), BE: −5.9 **(F)** PTE-MAPK1 (PDB: 8AOJ), BE: −6.1. **(G)** PTE-ESR1 (PDB: 7BAA), BE: −5.9. **(H)** PTE-MAPK8 (PDB: 2XRW), BE: −7.2. **(I)** PTE-PIK3CB (PDB: AF-P42338-F1), BE: −7.1. **(J)** PTE-RAF1 (PDB: 3IQU), BE: −5.6. BE: binding energy (kcal/mol).

Molecular docking analysis was conducted to evaluate the binding affinity of PTE with key potential targets. Based on the PPI network analysis and pathway enrichment results, ten core targets including SRC (PDB ID: 1FMK), HSP90AA1 (PDB ID: 5J80), PIK3CA (PDB ID: 9ASF), ESR1 (PDB ID: 7BAA), PIK3CB (PDB ID: AF-P42338-F1), PTPN11 (PDB ID: 3ZM1), EGFR (PDB ID: 8A27), MAPK1 (PDB ID: 8AOJ), MAPK8 (PDB ID: 2XRW), and RAF1 (PDB ID: 3IQU) were selected as receptors for docking with PTE to validate potential interactions. The docking results (Supplementary Table S6) showed binding energies ranging from −7.4 to −5.6 kcal/mol, indicating favorable binding affinity. Various interactions were observed between PTE and target proteins, including conventional hydrogen bonds, carbon-hydrogen bonds, electrostatic interactions (Pi-Cation and Pi-Anion), hydrophobic interactions (Alkyl, Pi-alkyl, Van der Waals), and π-π stacking (Figure 4). These results provide a detailed understanding of the interaction patterns between PTE and core targets, laying a foundation for its potential therapeutic effects in LIRI.

**FIGURE 5:**
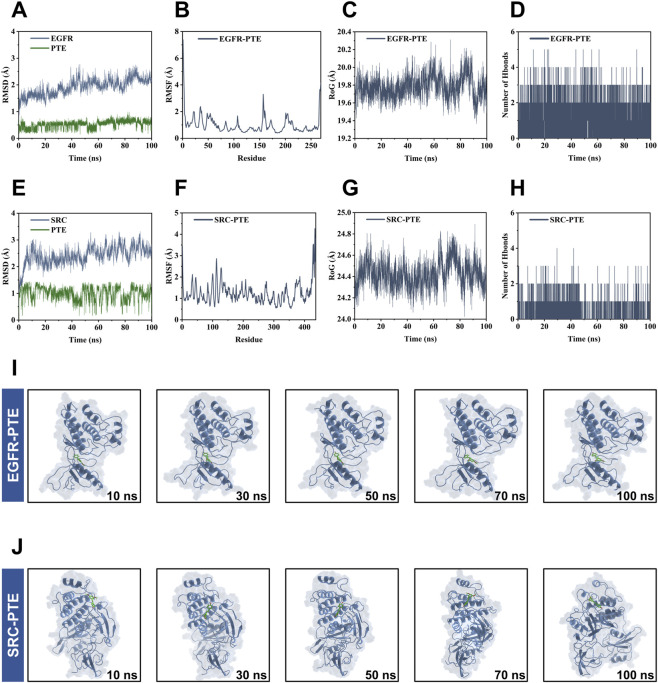
Molecular dynamics simulation of PTE binding to EGFR and SRC. **(A–D)** Time-dependent plots of RMSD, RMSF, RoG, and hydrogen bond numbers for the EGFR-PTE complex during a 0–100 ns MD simulations. **(E–H)** Time-dependent plots of RMSD, RMSF, RoG, and number of hydrogen bonds for the SRC-PTE complex during a 0–100 ns MD simulations. **(I)** Conformational changes of the EGFR-PTE complex during MD simulations. **(J)** Conformational changes of the SRC-PTE complex during MD simulations. MD: molecular dynamics, RMSD: root mean square deviation, RMSF: root mean square fluctuation, RoG: radius of gyration, Hbonds: conventional hydrogen bonds.

Molecular dynamics (MD) simulations were subsequently performed to assess the stability of PTE-target complexes. Based on PPI network, molecular docking, and mechanisms of IR injury, EGFR (with the lowest docking energy) and SRC (with the highest degree in the PPI network) were selected for simulation, both of which have been shown to modulate apoptosis or inflammation in IR injury (Li et al., 2022a; Lv et al., 2024; Oyaizu et al., 2012; Wang et al., 2022). The results of several key MD parameters including the root mean square deviation (RMSD), the root mean square fluctuation (RMSF), the radius of gyration (RoG), and number of hydrogen bonds (Hbonds), demonstrate that both the PTE-EGFR and PTE-SRC complexes maintained relatively stable conformations with strong intermolecular interactions (Figure 5). Furthermore, MM/PBSA calculations revealed the binding energies of PTE with target proteins and the contribution of the top 10 amino acid residues to the binding energy (Supplementary Tables S7 and S8). Collectively, these MD results indicate that PTE forms stable and robust interactions with core targets, suggesting that PTE may sustain sufficient *in vivo* activity and exert prolonged biological effects to mitigate LIRI.

### 2.4 PTE inhibits IR-induced apoptosis and inflammation in rat lung tissues and modulates expression of potential core targets

**FIGURE 6:**
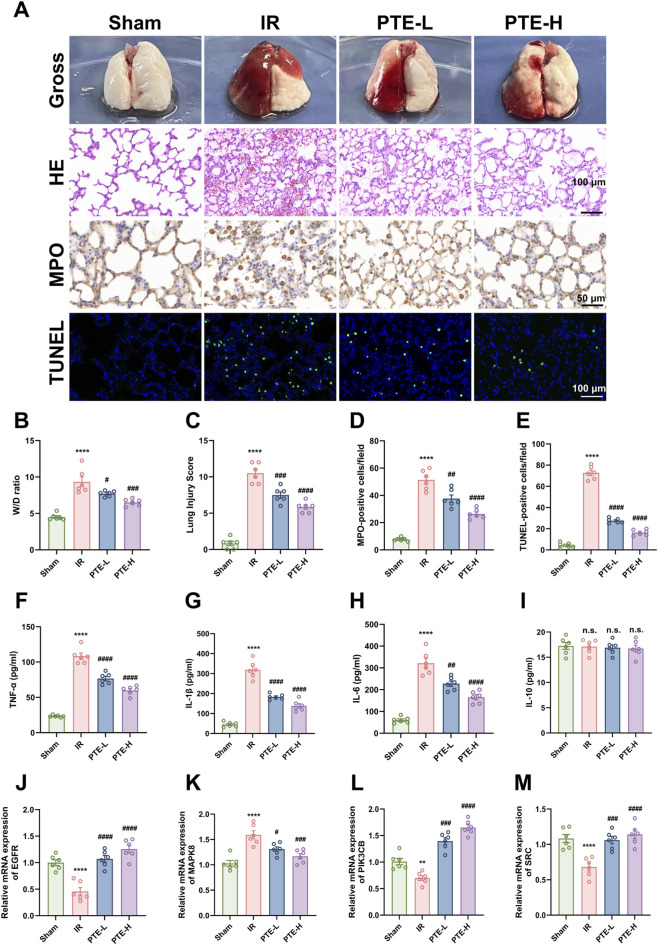
PTE mitigates IR-induced lung injury in rats. **(A)** Representative images showing gross morphology, HE staining, MPO immunohistochemistry (IHC), and TUNEL staining of rat lungs following 1 h of left hilar clamping and 2 h of reperfusion. PTE was administered as a single intraperitoneal injection at the onset of reperfusion (30 mg/kg for the low-dose group, PTE-L; 60 mg/kg for the high-dose group, PTE-H). The Sham group underwent no hilar occlusion, whereas the IR group received an equivalent volume of saline. **(B)** Pulmonary edema after reperfusion was assessed by the lung W/D ratio. **(C–E)** Histopathological damage was quantitatively evaluated using lung injury score, the number of MPO-positive cells, and the number of TUNEL-positive cells. **(F–I)** Concentrations of TNF-α, IL-1β, IL-6, and IL-10 in lung tissue homogenates were measured by ELISA. **(J–M)** Relative mRNA expression levels of EGFR, MAPK8, PIK3CB and SRC were determined by qRT-PCR. HE staining: scale bar = 100 μm; MPO staining: scale bar = 50 μm; TUNEL staining: scale bar = 100 μm. Data are presented as the mean ± SD (n = 6). ^*^ *P* < 0.05, ^**^ *P* < 0.01, ^***^ *P* < 0.001, ^****^ *P* < 0.0001, vs. Sham group; ^#^ *P* < 0.05, ^##^ *P* < 0.01, ^###^ *P* < 0.001, ^####^ *P* < 0.0001, vs. IR group; n.s = non-significant. PTE: pterostilbene, IR: ischemia-reperfusion, W/D: wet to dry, HE: hematoxylin and eosin, MPO: myeloperoxidase, TUNEL: terminal deoxynucleotidyl transferase dUTP nick end labeling, ELISA: enzyme-linked immunosorbent assay, qRT-PCR: quantitative real-time polymerase chain reaction.

To verify the therapeutic role of PTE in LIRI, a stable and reproducible rat left hilar clamping model was established (Supplementary Figure S1). Rats received PTE at doses of 30 mg/kg (PTE-L) or 60 mg/kg (PTE-H), or an equivalent volume of saline as control, at the onset of reperfusion. HE staining revealed severe pulmonary capillary congestion, interstitial edema, inflammatory cell infiltration, and alveolar wall thickening in the IR group compared tothe Sham group. These pathological changes were markedly alleviated in PTE-treated groups, resulting in significantly lower lung injury scores (Figures 6A,C). The lung wet-to-dry (W/D) weight ratio, a reliable indicator of pulmonary edema and vascular permeability, was substantially elevated in the IR group and effectively reduced by PTE treatment (Figure 6B). Similarly, more MPO-positive cells (an indicator of neutrophil infiltration) with brown spots and TUNEL-positive cells (reflecting apoptosis) with green fluorescence were observed in the IR group than in the Sham group, which were markedly reduced by PTE administration (Figures 6A,D,E). In addition, assessment of pulmonary cytokines further confirmed the anti-inflammatory effects of PTE. TNF-α, IL-1β, and IL-6 levels were markedly elevated in IR lungs and attenuated by PTE, whereas the level of IL-10 remained unchanged (Figures 6F–I). In conclusion, these findings indicate that PTE, particularly at the higher dose (PTE-H), effectively mitigates IR-induced lung injury, apoptosis, and inflammation.

Furthermore, based on network pharmacology and molecular docking results, EGFR, MAPK8, PIK3CB, and SRC were selected for *in vivo* validation by qPCR (Figures 6J–M), given their potential involvement in LIRI through regulatory effects on cell survival, stress responses, and inflammation. Compared to the Sham group, the mRNA expression levels of EGFR, PIK3B and SRC were substantially reduced in the IR group, while MAPK8 mRNA expression was significantly elevated. Notably, PTE treatment reversed these alterations in a dose-dependent manner. These results suggest that modulation of these core targets may underlie the protective effects of PTE against LIRI.

### 2.5 PTE ameliorates apoptosis and inflammation in the OGD/R cell model and modulates the expression of potential core targets

**FIGURE 7:**
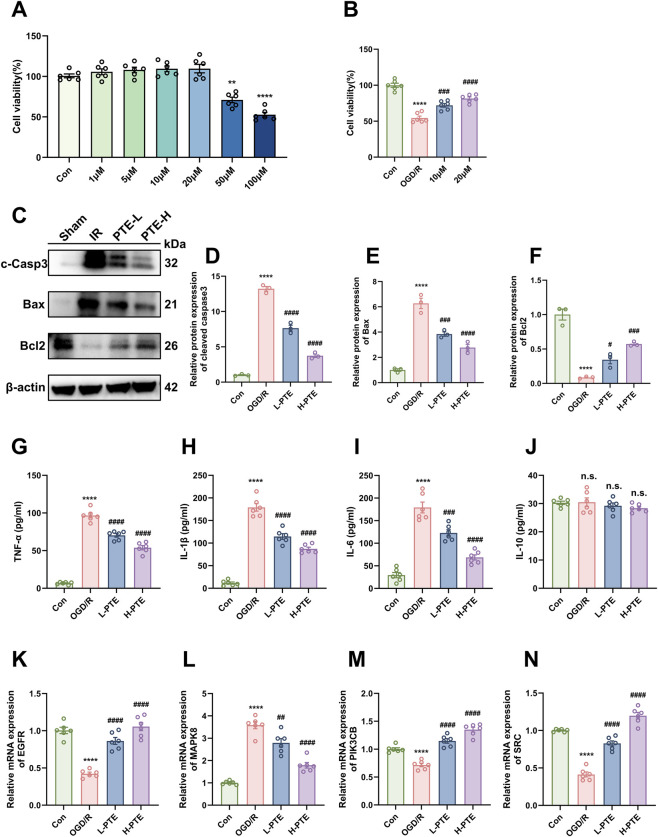
PTE protects against OGD/R-induced injury in BEAS-2B cells. **(A)** Cell viability after 24 h treatment with various concentrations of PTE under normoxic conditions, measured by CCK-8 assay. **(B)** Cell viability after OGD/R procedures (6 h OGD and 4 h R). PTE was applied at the onset of reoxygenation at final concentrations of 10 µM (L-PTE) and 20 µM (H-PTE). Control cells were maintained under normoxia, while the OGD/R group received an equal volume of PBS. **(C–F)** Western blotting analysis of pro-apoptotic proteins (cleaved Caspase-3 and Bax) and the anti-apoptotic protein Bcl-2. **(G–J)** Concentrations of TNF-α, IL-1β, IL-6, and IL-10 in cell culture supernatants measured by ELISA. **(K–N)** Relative mRNA expression levels of EGFR, MAPK8, PIK3CB, and SRC. Data are presented as the mean ± SD (n = 3 or n = 6). ^*^ *P* < 0.05, ^**^ *P* < 0.01, ^***^ *P* < 0.001, ^****^ *P* < 0.0001, vs. Con group; ^#^ *P* < 0.05, ^##^ *P* < 0.01, ^###^ *P* < 0.001, ^####^ *P* < 0.0001, vs. OGD/R group; n.s = non-significant. BEAS-2B: human bronchial epithelial cell line 2B, OGD/R: oxygen-glucose deprivation/reoxygenation, CCK-8: cell counting kit-8, PBS: phosphate-buffered saline, c-Casp3: cleaved Caspase-3, ELISA: enzyme-linked immunosorbent assay.

As illustrated in Figure 7A, treatment of BEAS-2B cells with 1–20 μM PTE for 24 h under normoxic conditions did not significantly affect cell viability compared to the control group. However, cell viability was markedly reduced at 50 and 100 μM, likely due to the accumulation of cytotoxicity. Therefore, 10 and 20 μM of PTE were selected for further experiments. In the OGD/R model, PTE dose-dependently restored the reduced cell viability induced by OGD/R (Figure 7B). Additionally, to determine whether the protective effects of PTE were associated with apoptosis, the protein levels of cleaved Caspase-3, Bcl-2, and Bax were evaluated by Western blotting (Figures 7C–F). The relative expression levels of pro-apoptotic proteins cleaved Caspase-3 and Bax were significantly increased in the OGD/R group compared with the Control group, while the relative expression level of antiapoptotic protein Bcl-2 was significantly reduced. However, PTE administration ameliorated these changes compared with the OGD/R group. Similarly, we measured the levels of inflammation-related cytokines (Figures 7G–J) and the mRNA expression of core targets (Figures 7K–N) *in vitro*, and the findings were generally consistent with those observed *in vivo*. These data suggest that PTE may exert cytoprotective effects by alleviating apoptosis and inflammation in the OGD/R model, which corroborates the findings *in vivo*, thus providing new evidence for the potential of PTE in the treatment of LIRI.

### 2.6 PTE mitigates LIRI-induced apoptosis and inflammation via the PI3K/AKT and JNK/c-Jun signaling pathways

**FIGURE 8:**
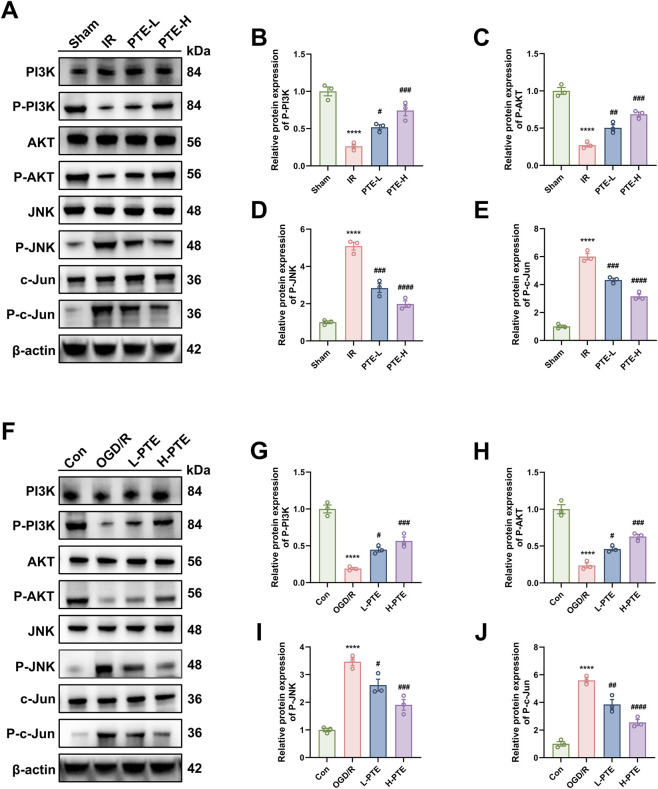
Effects of PTE on the PI3K/AKT and JNK/c-Jun signaling pathways. **(A–E)** *In vivo*, the relative expression levels of phosphorylated PI3K, AKT, JNK, and c-Jun proteins. **(F–J)** *In vitro*, the relative expression levels of phosphorylated PI3K, AKT, JNK, and c-Jun proteins. Data are presented as the mean ± SD (n = 3). ^*^ *P* < 0.05, ^**^ *P* < 0.01, ^***^ *P* < 0.001, ^****^ *P* < 0.0001, vs. Sham group or Con group; ^#^ *P* < 0.05, ^##^ *P* < 0.01, ^###^ *P* < 0.001, ^####^ *P* < 0.0001, vs. IR group or OGD/R group; n.s = non-significant. MAPK8 corresponds to JNK1, while JNK in Western blotting represents the total expression of JNK1/2/3. LIRI: lung ischemia-reperfusion injury.

**FIGURE 9:**
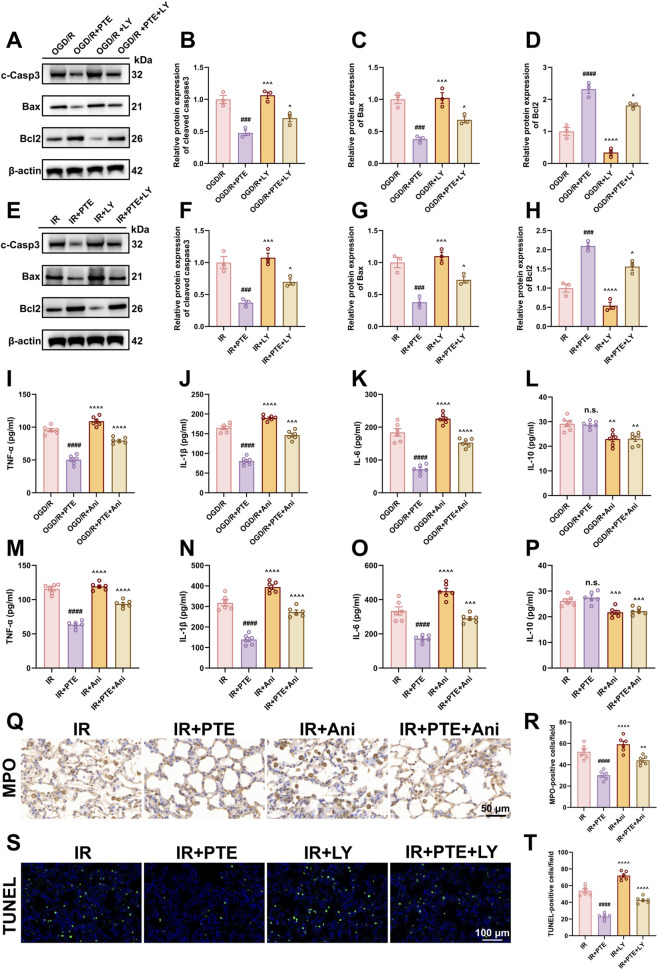
PTE confers anti-apoptotic and anti-inflammatory protection against LIRI in a PI3K/AKT and JNK/c-Jun dependent manner. In this section, the PI3K inhibitor (LY294002) and the JNK agonist (Anisomycin) were used to further investigate the role of these two signaling pathways in PTE-mediated protection against LIRI. PTE was administered at a high dose (60 mg/kg, i.p. *in vivo*; 20 µM *in vitro*) at the start of reperfusion or reoxygenation. Both LY294002 (0.3 mg/kg via tail vein injection *in vivo*; 10 µM *in vitro*) and Anisomycin (15 mg/kg, i.p. *in vivo*; 20 μg/mL *in vitro*) were given 30 min prior to modeling. **(A–H)** *In vitro* and *in vivo*, Western blotting analysis of pro-apoptotic proteins cleaved Caspase-3 and Bax and the anti-apoptotic protein Bcl-2. **(I**–**P)** *In vitro* and *in vivo*, concentrations of TNF-α, IL-1β, IL-6, and IL-10 in cell culture supernatants or tissue homogenates were measured by ELISA. **(Q,R)** Representative images of MPO immunohistochemical staining and the quantitative analysis. **(S,T)** Representative images of TUNEL staining and the quantitative analysis. MPO staining: scale bar = 50 μm; TUNEL staining: scale bar = 100 μm. Data are presented as the mean ± SD (n = 3 or n = 6). ^#^ *P* < 0.05, ^##^ *P* < 0.01, ^###^ *P* < 0.001, ^####^ *P* < 0.0001, vs. IR group or OGD/R group; ^^^ *P* < 0.05, ^^^^ *P* < 0.01, ^^^^^ *P* < 0.001, ^^^^^^ *P* < 0.0001, vs. IR + PTE or OGD/R + PTE group; n.s = non-significant. c-Casp3: cleaved Caspase-3, LY: LY294002, Ani: Anisomycin, i.p.: intraperitoneal injection.

**FIGURE 10:**
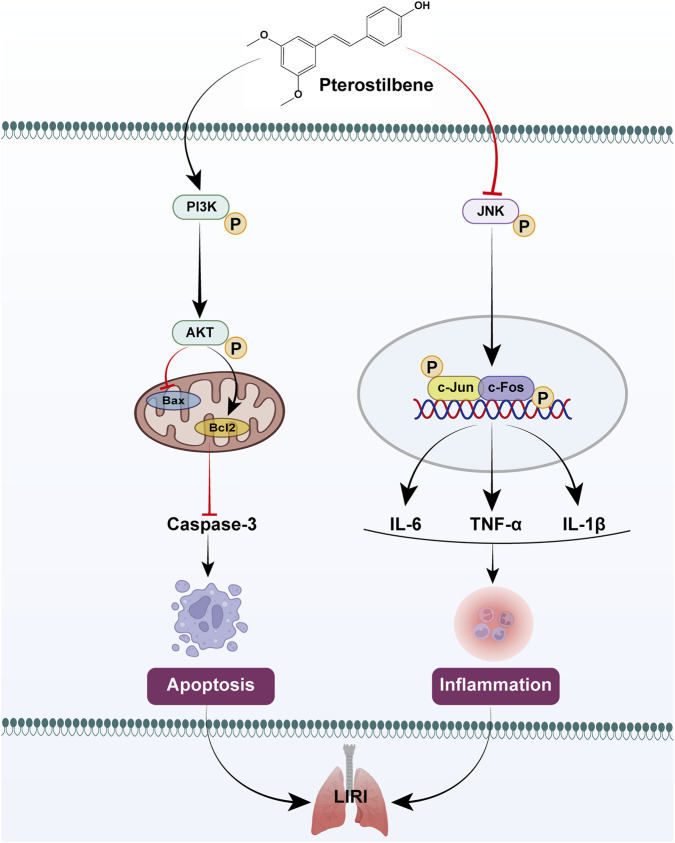
Proposed mechanism of PTE in alleviating LIRI.

Based on the core targets and pathways identified by network pharmacology analysis, together with evidence from relevant literature, we hypothesize that PTE may alleviate LIRI by exerting anti-apoptotic effects via the PI3K/AKT pathway and anti-inflammatory effects via the JNK/c-Jun pathway. We first assessed the activation of the PI3K/AKT and JNK/c-Jun pathways through Western blot (Figure 8). Compared to the Sham and Control groups, IR or OGD/R groups significantly reduced PI3K/AKT phosphorylation and increased JNK/c-Jun phosphorylation. Notably, PTE treatment dose-dependently reversed these effects. To further clarify whether the anti-apoptotic and anti-inflammatory effects of PTE are mediated through the PI3K/AKT and JNK/c-Jun pathways, we employed a PI3K inhibitor (LY294002, LY) and a JNK activator (Anisomycin, Ani) in subsequent experiments. Both agents effectively modulated their respective targets and attenuated PTE’s effects on pathway activity (Supplementary Figure S4). Subsequently, we measured apoptosis- and inflammation-related markers. LY reversed the anti-apoptotic effects of PTE, evidenced by upregulation of cleaved-Caspase3 and Bax, downregulation of Bcl-2 and an increased number of TUNEL-positive cells (Figures 9A–H,S,T). Ani counteracted the anti-inflammatory effects of PTE, as increased indicated by elevated levels of TNF-α, IL-1β, and IL-6, reduced IL-10 levels and an increased number of MPO-positive cells (Figures 9I–R). Collectively, these findings suggest that the protective effects of PTE against LIRI are mediated, at least in part, by activation of the PI3K/AKT anti-apoptotic pathway and inhibition of the JNK/c-Jun inflammatory pathway, as illustrated in the schematic diagram (Figure 10).

## 3 Discussion

This study investigated the potential therapeutic targets and signaling pathways of PTE in treating LIRI by integrating network pharmacology, molecular docking, molecular dynamics simulations, and *in vitro* and *in vivo* experimental validation.

PTE is a natural dimethoxy analog of resveratrol (RSV), predominantly found in blueberries, peanuts, and grapes (Nagarajan et al., 2022). Accumulating evidence indicates that PTE exhibits diverse pharmacological properties, including antioxidant, anti-inflammatory, antitumor, neuroprotective, and metabolic regulatory effects (Li et al., 2024a; Teng et al., 2021; Yang et al., 2020; Zhao et al., 2021), making it a highly promising therapeutic agent for clinical application. These properties render PTE a promising candidate for the prevention and treatment of various conditions, including diabetes, cancer, cardiovascular diseases, neurodegenerative disorders, and age-related pathologies. Notably, PTE has been demonstrated to confer protective effects against ischemia-reperfusion injury in several organs, such as the heart (Wu et al., 2017; Yu et al., 2017), brain (Ban et al., 2024; Yang et al., 2023), liver (Shi et al., 2022), and kidney (Gao et al., 2018). However, its potential role in LIRI and the underlying molecular mechanisms remain unexplored. Interestingly, this study provides the first evidence that PTE confers dose-dependent pharmacological protection against LIRI in a rat IR model and in a BEAS-2B cell OGD/R model. Furthermore, from a pharmacokinetic perspective, the concentrations of PTE used in this study show translational feasibility and advantages. Compared with RSV, PTE displays superior oral bioavailability and metabolic stability, largely due to the presence of two methoxy groups, which enhance lipophilicity and membrane permeability (Lin et al., 2020). Rodent studies have demonstrated that the oral bioavailability of PTE can reach up to 80%, whereas resveratrol remains below 20% (Ali et al., 2025; Peng et al., 2018), highlighting its pharmacokinetic superiority and enhanced therapeutic potential. Furthermore, PTE exhibits an excellent safety profile, with no observable toxicity in mice at doses as high as 3,000 mg/(kg·day) (Ruiz et al., 2009), and is well tolerated in humans at doses up to 250 mg/day (Riche et al., 2013). These findings indicate that clinically safe and effective plasma or tissue concentrations of PTE can be achieved through intravenous or oral administration, further underscoring its considerable potential for real-world pharmaceutical applications.

Apoptosis plays a pivotal role in the pathogenesis of LIRI (Capuzzimati et al., 2022; Fischer et al., 2000b; Yu et al., 2024). During reperfusion, the sudden restoration of oxygen supply provokes excessive reactive oxygen species (ROS) production, mitochondrial dysfunction, and calcium overload, which collectively activate apoptotic signaling cascades (den Hengst et al., 2010; Fischer et al., 2000a). Activation of both intrinsic (mitochondrial cytochrome c release and Caspase-3 activation) and extrinsic (death receptor-mediated) pathways leads to widespread epithelial and endothelial cell apoptosis (Derakhshan et al., 2017; Lossi, 2022). The resulting cellular injury compromises the integrity of the alveolar-capillary barrier, aggravates pulmonary edema, and impairs gas exchange (Iske et al., 2021). Consequently, targeting apoptosis has emerged as a promising therapeutic strategy for mitigating LIRI and improve post-transplant pulmonary function. In this study, GO and KEGG enrichment analyses indicated a strong correlation between PTE’s potential targets and apoptotic processes, suggesting that it may represent one of the core mechanisms underlying the protective effects of PTE against LIRI. Experimental validation further supported this hypothesis. *In vivo*, PTE markedly reduced the number of TUNEL-positive cells in lung tissues following IR. *In vitro*, PTE increased the expression of the anti-apoptotic protein Bcl-2 while decreasing Bax and cleaved Caspase-3 expression. These findings align with previous reports (Pelles-Taskó et al., 2024; Yang et al., 2020; Zhang et al., 2012; Zhou et al., 2019) on the anti-apoptotic effects of PTE; however, the precise molecular mechanisms through which it modulates apoptosis in LIRI remain to be elucidated. Previous studies have shown that the p38 MAPK (Zhang et al., 2003), RIPK1 (Dong et al., 2022), FXR/LKB1 (Dong et al., 2021), and SIRT1/PGC-1α (Yang et al., 2021a) pathways play critical roles in regulating apoptosis during LIRI. Moreover, mesenchymal stromal cell-derived exosomes were shown to inhibit both intrinsic and extrinsic apoptotic pathways by delivering miR-21-5p, which targets PTEN and PDCD4 (Li et al., 2019). Based on core target identification, GO and KEGG pathway enrichment analyses, together with evidence from relevant literature, the PI3K/AKT pathway was identified as a key candidate mediating the anti-apoptotic effects of PTE. As is well known, the PI3K/AKT axis serves as a critical regulator of cell survival, proliferation, and metabolism (Yu and Cui, 2016). Under most physiological and pathological conditions, the PI3K/AKT pathway acts as a key cell survival signal, inhibiting apoptosis through multiple mechanisms (Franke et al., 2003; Glaviano et al., 2023; Hoxhaj and Manning, 2020). Upon activation, PI3K catalyzes the formation of PIP3, leading to AKT activation, which in turn suppresses apoptosis by inactivating pro-apoptotic proteins (Bad, Bax), upregulating anti-apoptotic molecules (Bcl-2, Bcl-xL), inhibiting Caspase-9/3 activation, and repressing FoxO-mediated transcription of pro-apoptotic genes such as Bim and FasL. However, under certain pathological conditions, sustained or aberrant activation of the PI3K/AKT pathway can paradoxically induce apoptosis (He et al., 2021; Park et al., 2015; Zhao et al., 2017). Notably, our results (Figures 8, 9) indicated that PTE treatment activated the PI3K/AKT pathway, and the anti-apoptotic effects of PTE were attenuated by LY294002 (a PI3K inhibitor). Taken together, these findings suggest that PTE inhibits LIRI-induced apoptosis, potentially via activation of PI3K/AKT signaling.

In addition to various forms of cell death including apoptosis, inflammatory is widely recognized as another key contributor to ischemia-reperfusion injury (Jeon et al., 2024; Li and Nie, 2024; Ta et al., 2024). Briefly, the sudden restoration of blood flow triggers oxidative stress, endothelial cell activation, and the release of damage-associated molecular patterns (DAMPs) from injured cells. These DAMPs signals bind to corresponding receptors on various cell types, initiating the activation and recruitment of innate immune cells, complement activation, and the secretion of pro-inflammatory cytokines and chemokines. This inflammatory cascade further exacerbates lung injury and may ultimately lead to graft dysfunction or failure. Our ELISA data (Figures 6F–I, 7G–J), demonstrated that PTE markedly reduced the production of pro-inflammatory cytokines, including TNF-α, IL-1β, and IL-6 both *in vivo* and *in vitro*. However, no significant differences were observed in the levels of the anti-inflammatory cytokine IL-10 among the groups, which may be attributed to the specific time window of detection in the IR model, the complex regulation of the immune system, and the relatively short half-life of IL-10 (Saraiva and O'Garra, 2010; Thomassen et al., 1996; Yeung et al., 2025). These findings are consistent with previous studies, suggesting that PTE exerts anti-inflammatory effects to alleviate LIRI. Similar to our screening strategy for apoptosis-related pathways, we identified the MAPK8 (JNK1)/c-Jun signaling pathway as a potential mediator of PTE’s anti-inflammatory effects, which was subsequently validated experimentally. The JNK/c-Jun signaling pathway is a crucial stress-responsive kinase cascade that governs cellular responses to oxidative stress, cytokines stimulation, and environmental insults (Yan et al., 2024). Under pathological conditions, JNK/c-Jun is regarded as a pro-inflammatory signaling pathway (Graczyk, 2013; Hammouda et al., 2020; Zeke et al., 2016). Specifically, activation of JNK promotes c-Jun phosphorylation and the formation of the AP-1 complex, which translocates to the nucleus to promote transcription of pro-inflammatory genes such as TNF-α, IL-1β, IL-6, COX-2, and iNOS. Consistent with this predicted mechanism, our data (Figures 8, 9) showed that PTE inhibited the JNK/c-Jun pathway, and Anisomycin (a JNK activator) reversed its anti-inflammatory effects. In summary, our findings suggest that the anti-inflammatory effects of PTE in LIRI are likely mediated through inhibition of the JNK/c-Jun signaling pathway.

Collectively, PTE, a natural compound with favorable oral bioavailability, metabolic stability, and a strong safety profile, provides distinct advantages and substantial translational potential in current LIRI therapeutic strategies owing to its multi-target regulation of key pathological mechanisms (apoptosis and inflammation) in this study. Nevertheless, several limitations should be acknowledged. First, the network pharmacology analysis relies on the accuracy of public databases, and molecular docking and MD simulations are influenced by algorithmic assumptions and parameter settings, which may introduce uncertainty into target identification and interaction prediction. Second, while our results support that PTE alleviates LIRI through the PI3K/AKT and JNK/c-Jun pathways, the direct upstream molecular mechanisms by which PTE regulates these pathways, the contributions and crosstalk of other targets and signaling pathways, as well as the synergistic interplay between inflammation and apoptosis in exacerbating injury, remain unclear and require further investigation. Moreover, given the broad bioactivity of PTE, it is still unknown whether the observed benefits are predominantly mediated through the identified pathways or reflect general pharmacological effects. Third, this study relied on a single BEAS-2B cell line *in vitro* and a rat hilar occlusion model *in vivo*, which may not fully replicate the true clinical conditions of lung transplantation. Therefore, future research need to further validate these findings using diverse cell lines and large animal transplant or *ex vivo* perfusion models. Finally, addressing issues such as dose equivalence, delivery method, optimal timing of administration, bioavailability, and potential toxicity of PTE in the context of clinical lung transplantation is crucial for its clinical translation.

In summary, this study is the first to extend the protective effects of PTE to LIRI, systematically elucidating its dual mechanism of inhibiting apoptosis and inflammation through the regulation of the PI3K/AKT and JNK/c-Jun pathways. These findings offer valuable insights into the translational potential of PTE for the treatment of LIRI.

## 4 Materials and methods

### 4.1 Animals and lung left hilar clamping model

Specific-pathogen-free (SPF) adult male Sprague-Dawley rats (200–250 g) were obtained from the Laboratory Animal Center of Xi’an Jiaotong University Health Science Center (Xi’an, China). The animals were housed under controlled conditions (temperature 21 °C–25 °C, relative humidity 60%–70%, 12 h light/dark cycle) with free access to food and water. All experimental procedures were conducted in accordance with the Guide for the Care and Use of Laboratory Animals (NIH, United States) and approved by the Animal Ethics Committee of the First Affiliated Hospital of Xi’an Jiaotong University.

The rat left hilar clamping model of lung I/R was established according to the procedure as previously described (Lee et al., 2021; Saito et al., 2019; Tanaka et al., 2016). Briefly, rats were anesthetized with sodium pentobarbital (120 mg/kg, i.p.) and mechanically ventilated (tidal volume 10 mL/kg, respiratory rate 70 breaths/min, PEEP 2 cmH_2_O, FiO_2_ = 1.0). Heparin (50 IU) was injected via the jugular vein prior to left thoracotomy at the fifth intercostal space. The left pulmonary hilum was occluded with an atraumatic microvascular clamp for 60 min to induce ischemia, during which the tidal volume was reduced to 6 mL/kg. Reperfusion was initiated by removing the clamp and continued for 120 min. Rats in the sham group received the same procedure without hilar occlusion.

### 4.2 Cell culture and OGD/R model

Although the human bronchial epithelial (BEAS-2B) cells may not fully replicate the characteristics of alveolar epithelial cells, they remain the only non-cancerous human lung cell line available for research. Additionally, BEAS-2B cells are more sensitive to oxidative stress, inflammation, and cell death compared to alveolar epithelial and endothelial cells (Berggren-Nylund et al., 2023; Saren et al., 2021), making them particularly suitable for simulating the early mechanisms of LIRI. Meanwhile, the widespread application of BEAS-2B cells in the literature (Bojic et al., 2024; Zhang et al., 2025; Zhao et al., 2023; Zhu et al., 2025) further justifies their selection as the *in vitro* model for this study. Our BEAS-2B cells were obtained from American Type Culture Collection (ATCC, USA) and cultured in high-glucose Dulbecco’s modified Eagle’s medium (DMEM, PM150210, Procell, China), supplemented with 10% fetal bovine serum (FBS, S711-001S, Lonsera, UY) and 1% penicillin-streptomycin (HY-K1006, MCE, United States) in a 5% CO_2_ cell incubator at 37 °C and 95% relative humidity.

To more accurately mimic ischemia-reperfusion injury *in vitro*, an oxygen-glucose deprivation/reoxygenation (OGD/R) model was established as previously described (Zhao et al., 2022; Zhuang et al., 2025). Briefly, for OGD induction, the culture medium was replaced with glucose- and serum-free DMEM (PM150270, Procell, China), and cells were incubated in a hypoxic chamber (1% O_2_, 5% CO_2_, 94% N_2_) at 37 °C for 6 h. Reoxygenation was subsequently achieved by returning the cells to normoxic conditions in complete medium for 4 h. Control cells were cultured under normoxic conditions throughout the experiment.

### 4.3 Grouping and drug administration

Pterostilbene (PTE, HY-N0828), LY294002 (LY, HY-10108), and Anisomycin (Ani, HY-18982) were all purchased from MedChemExpress (MCE, United States), and dissolved in dimethyl sulfoxide (DMSO, D8371, Solarbio, China) before dilution to the desired concentrations for subsequent experiments. Dosage and administration regimen were primarily determined based on previous studies (He and Tong, 2022; He et al., 2023; Li et al., 2022b; Park et al., 2018; Tian et al., 2022; Yang et al., 2021b; Zhang et al., 2020; Zhang et al., 2024). Additionally, the safety of the experimental dose *in vivo* was further evaluated through preliminary experiments and toxicity assessments, including observations of animal behavior, liver and kidney function tests, and histopathological examinations.

*In vivo*, rats were randomly assigned to four groups (n = 6): Sham, IR model (IR), IR treated with 30 mg/kg PTE (PTE-L), and IR treated with 60 mg/kg PTE (PTE-H). At the onset of reperfusion, PTE was administered via intraperitoneal injection: 30 mg/kg for the PTE-L group and 60 mg/kg for the PTE-H group. Sham and IR groups received an equivalent volume of physiological saline. *In vitro*, cell viability of BEAS-2B cells following PTE treatment (0, 1 μM, 5 μM, 10 μM, 20 μM, 50 μM, 100 μM) was evaluated using the CCK-8 assay. To further research the effect of PTE on cell viability in OGD/R cell models, four groups were set up (n = 6): Control (Con), OGD/R model (OGD/R), OGD/R treated with 10 μM PTE (L-PTE), and OGD/R treated with 20 μM PTE (H-PTE). Similar to the *in vivo* experiments, drug interventions were applied at the onset of reoxygenation, with PTE added to the culture medium at final concentrations of 10 μM (L-PTE) and 20 μM (H-PTE), whereas Con and OGD/R groups received an equal volume of phosphate-buffered saline (PBS).

The LY inhibitor experiment was conducted to validate the necessity of the PI3K/AKT pathway in mediating the anti-apoptotic effects of PTE. Animals and cells were divided into four groups (n = 6): model group (IR or OGD/R), PTE group (IR + PTE or OGD/R + PTE), LY group (IR + LY or OGD/R + LY), and PTE + LY group (IR + PTE + LY or OGD/R + PTE + LY). PTE was administered according to the previously established high-dose regimen. LY pre-treatment was performed 30 min before modeling: rats received a tail vein injection of 0.3 mg/kg, or cells were treated with a final concentration of 10 μM in culture medium. Similarly, the Ani activator experiment was conducted to verify the necessity of the JNK/c-Jun pathway in mediating the anti-inflammatory effects of PTE. Animals and cells were divided into four groups (n = 6): model group (IR or OGD/R), PTE group (IR + PTE or OGD/R + PTE), Ani group (IR + Ani or OGD/R + Ani), and PTE + Ani group (IR + PTE + Ani or OGD/R + PTE + Ani). PTE was administered following the high-dose regimen. Ani pre-treatment was performed 30 min prior to modeling: rats received an intraperitoneal injection of 15 mg/kg, or cells were treated with a final concentration of 20 μg/mL in culture medium.

### 4.4 Lung wet/Dry weight ratio (W/D)

The severity of pulmonary edema was evaluated using the lung wet/dry (W/D) weight ratio. The left upper lobe was removed immediately after 2 h of reperfusion and weighed to obtain the wet weight. The tissue was then dried in an oven at 65 °C for 72 h to determine the dry weight, and the W/D ratio was calculated accordingly.

### 4.5 Hematoxylin and eosin (HE) staining

HE staining was performed to evaluate morphological changes in lung tissues. Following the LIRI procedure, lung samples were excised and fixed in 4% paraformaldehyde at 4 °C for 24 h. The tissues were then embedded in paraffin, sectioned at 4 µm thickness, and stained using a Hematoxylin-Eosin Staining Kit (G1003, Servicebio, Beijing, China). And then the sections were observed under a light microscope (Axioscan 7, Zeiss, Germany). Lung tissue damage was assessed in a blinded manner, following previously published methods (Matute-Bello et al., 2011; Zhao et al., 2023).

### 4.6 Immunohistochemistry (IHC)

Myeloperoxidase (MPO) immunohistochemistry was performed to assess pulmonary inflammation following ischemia-reperfusion (IR) injury. Briefly, paraffin-embedded lung tissues were sectioned (4 μm), deparaffinized, rehydrated, and subjected to antigen retrieval in citrate buffer (pH 6.0). Sections were then blocked with 5% BSA and incubated overnight at 4 °C with anti-MPO antibody (AF7494, 1:150, Beyotime, China). After incubation with an HRP-conjugated secondary antibody, staining was visualized using DAB substrate, and nuclei were counterstained with hematoxylin. MPO-positive cells were quantified in a blinded manner.

### 4.7 Terminal deoxynucleotidyl transferase dUTP nick end labeling (TUNEL) staining

TUNEL staining was conducted to evaluate cell apoptosis in lung tissues. At the end of the IR procedure, lung tissues were fixed in 4% paraformaldehyde, dehydrated through an ethanol gradient, embedded in paraffin, and sectioned at 4 µm. Sections were stained using a TUNEL assay kit (G3250, Promega, United States) following the manufacturer’s instructions and counterstained with DAPI. Apoptotic cells were observed under a fluorescence microscope (Zeiss, Germany) in a blinded manner to minimize potential bias.

### 4.8 Cell counting Kit-8 (CCK-8) assay

Cell viability was evaluated with the Cell Counting Kit-8 (CCK-8, C0005, TargetMol, United States) according to the guidelines of the manufacturer, employing a colorimetric method. Briefly, at the end of the treatment, 10 μL CCK-8 working solution was added to each well of a 96-well plate, which was then incubated for 1 h at 37 °C. Subsequently, the absorbance at 450 nm was measured using a microplate readerthe (Thermo Fisher, United States).

### 4.9 Network pharmacological analysis

#### 4.9.1 Target acquisition of PTE and LIRI

The two-dimensional (2D) molecular structure and CAS number of PTE were obtained from PubChem database (https://pubchem.ncbi.nlm.nih.gov/). Potential targets of PTE were collected from the Traditional Chinese Medicine Systems Pharmacology database (TCMSP, https://www.tcmsp-e.com/tcmsp.php), Swiss Target Prediction database (http://www.swisstargetprediction.ch) and PharmMapper database (http://lilab-ecust.cn/pharmmapper/). LIRI-associated targets were retrieved from the GeneCards database (https://www.genecards.org/), the Online Mendelian Inheritance in Man database (OMIM, http://www.omim.org/), and the Therapeutic Target Database (TTD, https://db.idrblab.net/ttd/). To standardize target names and transform gene names, the UniProt database (https://www.uniprot.org/) was employed. The common genes between PTE- and LIRI-related targets were identified as candidate targets through Venny 2.1.0 platform (https://bioinfogp.cnb.csic.es/tools/venny/index.html).

#### 4.9.2 Construction of protein-protein interaction (PPI) network and identification of core targets

The intersecting targets of PTE against LIRI (restricted to *Homo sapiens*) were imported into the STRING database (version 11.5, https://cn.string-db.org/) to construct the protein-protein interaction (PPI) network, with the minimum interaction score set at 0.900. The resulting network was visualized and analyzed using Cytoscape software (version 3.10.2, http://www.cytoscape.org/). The cytoHubba and Network Analyzer plugins were used to identify the core targets based on key topological parameters, including degree, betweenness centrality, closeness centrality, and topological coefficient.

#### 4.9.3 Enrichment analyses of gene ontology (GO) and kyoto encyclopedia of genes and genomes (KEGG) pathway

GO and KEGG enrichment analyses were performed using the DAVID database (https://david.ncifcrf.gov/). The top 10 or 30 significantly enriched terms (*P* < 0.05) in biological processes (BP), cellular components (CC), molecular functions (MF), and signaling pathways were visualized using an online bioinformatics platform (http://www.bioinformatics.com.cn).

#### 4.9.4 Establishment of the compound -target-pathway-disease (CTPD) network

To visualize and elucidate the complex associations among compound, targets, signaling pathways and disease, a CTPD network was constructed and visualized using Cytoscape software (version 3.10.2). In the network, compound, targets, pathways, and disease were represented as nodes, while their interactions were represented as edges.

### 4.10 Molecular docking

The three-dimensional (3D) structures of the target proteins were retrieved from the Protein Data Bank database (PDB, https://www.rcsb.org/) and were processed by removing water molecules, irrelevant protein chains, and original ligands using PyMOL 2.5.4 software. The 3D structure of PTE was downloaded from the PubChem database and optimized in Chem3D 22.0.0 software to obtain the lowest-energy energetic conformation. The prepared protein receptors and ligand molecules were then converted into PDBQT format for docking analysis. Finally, molecular docking was performed using AutoDock Vina 1.2.5, and the resulting complexes were visualized with PyMOL 2.5.4. The docking score represented the predicted binding affinity between the receptor and ligand, with lower scores indicating stronger interactions. Generally, a docking score lower than −6.0 kcal/mol was considered indicative of a strong binding affinity (Tang et al., 2021).

### 4.11 Molecular dynamics simulation

To further evaluate the stability and dynamic behavior of the interactions between PTE and core target proteins, molecular dynamics (MD) simulations were carried out using GROMACS 2025.3 software, following previously described protocols (Lei et al., 2022; Li et al., 2024b). The docking complex with the lowest binding energy for each target was selected as the initial conformation for MD simulation. The system was simulated under constant temperature (300 K) and pressure (1 bar) conditions. The Amber99sb-ildn force field was employed, and the system was solvated in a TIP3P water box. To ensure electrostatic neutrality, Na^+^ and Cl^−^ counterions were added. Energy minimization was performed using the steepest descent algorithm, followed by equilibration under the NVT and NPT ensembles (100,000 steps each) with a coupling constant of 0.1 ps for 100 ps. Subsequently, a 100 ns production run was conducted with a 2 fs integration step (5,000,000 steps in total). The trajectory data were analyzed to evaluate the structural stability and flexibility of the complexes based on the root mean square deviation (RMSD), root mean square fluctuation (RMSF), radius of gyration (RoG), and the number of conventional hydrogen bonds (H-bonds). The binding free energy (ΔG_bind)_ between PTE and target proteins was further estimated using the molecular mechanics Poisson-Boltzmann surface area (MM-PBSA) method (Kollman et al., 2000; Kumari et al., 2014).

### 4.12 Enzyme-linked immunosorbent assay (ELISA)

The levels of tumor necrosis factor TNF-α, IL-1β, IL-6, and IL-10 in cell culture supernatants or lung tissue homogenates were measured using ELISA kits (Elabscience, China) according to the manufacturers’ guidelines.

### 4.13 Quantitative real-time PCR (qRT-PCR)

Total RNA from lung tissues or cells was extracted using Triquick Reagent (R1100, Solarbio, China) following the manufacturer’s instructions. cDNA synthesis was performed using All-in-one RT SuperMix (AG0305-B, Sparkjade, China), and quantitative amplification was conducted with SYBR Green qPCR Mix (AH0104-B, Sparkjade, China) on an AB7500 Fast Real-Time System. Gene expression levels were normalized to GAPDH. Primers for GAPDH were obtained from Sangon Biotech (Shanghai, China; B661104-0001 and B661204-0001), and sequences of all other primers used in this study are provided in Supplementary Table S1.

### 4.14 Western blotting

Total protein of cells or tissues was extracted using RIPA lysis buffer (P0013B, Beyotime, China) supplemented with protease inhibitors (ST506, Beyotime, China) and phosphatase inhibitors (P1260, Solarbio, China). Protein concentrations were determined using a BCA assay kit (P0012, Beyotime, China). Proteins were separated by 4%–20% SDS-PAGE according to molecular weight, and then transferred onto PVDF membranes. The membranes were blocked with 5% nonfat dry milk in TBST for 1 h at room temperature, and were then incubated overnight at 4 °C with the following primary antibodies: cleaved Caspase-3 (1:1,000, 25128-1-AP, Proteintech, China), Bax (1:1,000, AF0120, Affinity, China), Bcl-2 (1:1,000, AF6139, Affinity, China), PI3K (1:1,000, AF6241, Affinity, China), p-PI3K (1:1,000, AF3242, Affinity, China), AKT (1:1,000, AF6261, Affinity, China), p-AKT (1:1,000, AF0016, Affinity, China), JNK(1:1,000, AF6318, Affinity, China), p-JNK(1:1,000, AF3318, Affinity, China), c-Jun (1:1,000, AF6090, Affinity, China), p-c-Jun (1:1,000, AF3095, Affinity, China) and β-actin (1:20,000, 81115-1-RR, Proteintech, China). The membranes were subsequently incubated for 1 h with goat anti-rabbit IgG-HRP (1:5,000, LF102, Epizyme, China). Protein bands were visualized using ECL reagent (SQ201, Epizyme, China) and quantified with ImageJ software (Java 1.8.0, NIH, United States).

### 4.15 Statistical analysis

All experiments were independently repeated at least three times and data are presented as the mean ± standard deviation (SD). Statistical analyses were performed with GraphPad Prism 10.1.2 software (San Diego, CA, United States). Normality of the data was assessed using the Shapiro-Wilk test before performing one-way analysis of variance (ANOVA), followed by Tukey’s *post hoc* test for multiple group comparisons. A *P* value < 0.05 was considered statistically significant.

## Data Availability

The original contributions presented in the study are included in the article/Supplementary Material, further inquiries can be directed to the corresponding author.
