# Supplementary material for: Pterostilbene attenuates lung ischemia-reperfusion injury: integrative insights from network pharmacology, molecular dynamics, and experimental validation
Source: Front Pharmacol. 2026 Apr 1;17:1747977. doi: 10.3389/fphar.2026.1747977 (PMC13079632; doi:10.3389/fphar.2026.1747977)
Supplement: Supplementary file 1 [file DataSheet1.pdf]

Support Information (SI)

Pterostilbene attenuates lung ischemia-reperfusion injury: integrative insights from network pharmacology, molecular dynamics, and experimental validation

Haotian Bai[1,2], Heng Zhao[1,2], Jinteng Feng[1,2], Hongyi Wang[1,2], Yixing Li[1,2], Zhe Chen[1,2], Bin He[1,2], Chi Wang[1,2], Rui Gao[3], Rui Zhao[1,2], Shan Gao[1,2], Guangjian Zhang[1,2]*

[1] Department of Thoracic Surgery, The First Affiliated Hospital of Xi'an Jiaotong University, Xi'an, 710061, P.R. China
[2] Key Laboratory of Enhanced Recovery After Surgery of Integrated Chinese and Western Medicine, Administration of Traditional Chinese Medicine of Shaanxi Province, The First Affiliated Hospital of Xi'an Jiaotong University, Xi'an, 710061, P.R. China
[3] Department of Nuclear Medicine, The First Affiliated Hospital of Xi'an Jiaotong University, Xi'an, 710061, P.R. China

* Correspondence should be addressed to:
Guangjian Zhang, PhD, E-mail: michael8039@xjtu.edu.cn

Contents

1. Supplementary Figures 1

Figure S1 1

Figure S2 2

Figure S3 3

Figure S4 4

2. Supplementary Tables 6

Table S1 6

Table S2 7

Table S3 8

Table S4 9

Table S5 10

Table S6 11

Table S7 12

Table S8 13

1. Supplementary Figures

Figure S1

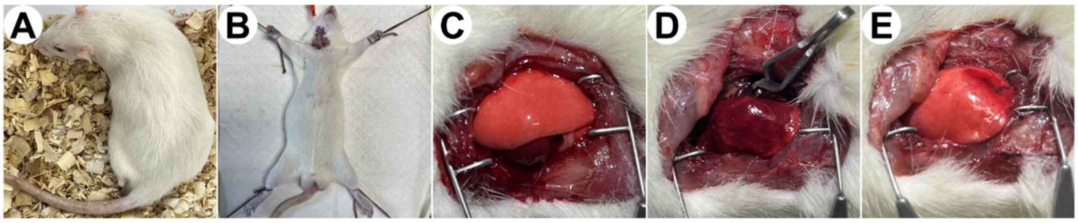

Figure S1. Establishment of a left lung hilar clamping IR model in rats. (**A**) Induction and maintenance of anesthesia (**B**) Endotracheal intubation and ventilation (**C**) Thoracotomy and exposure of the left pulmonary hilum (**D**) Ischemia phase: occlusion of the pulmonary hilum with an atraumatic vascular clip **(E)** Reperfusion phase: removal of the clip to restore blood flow. IR: ishchemia-reperfusion.

Figure S2

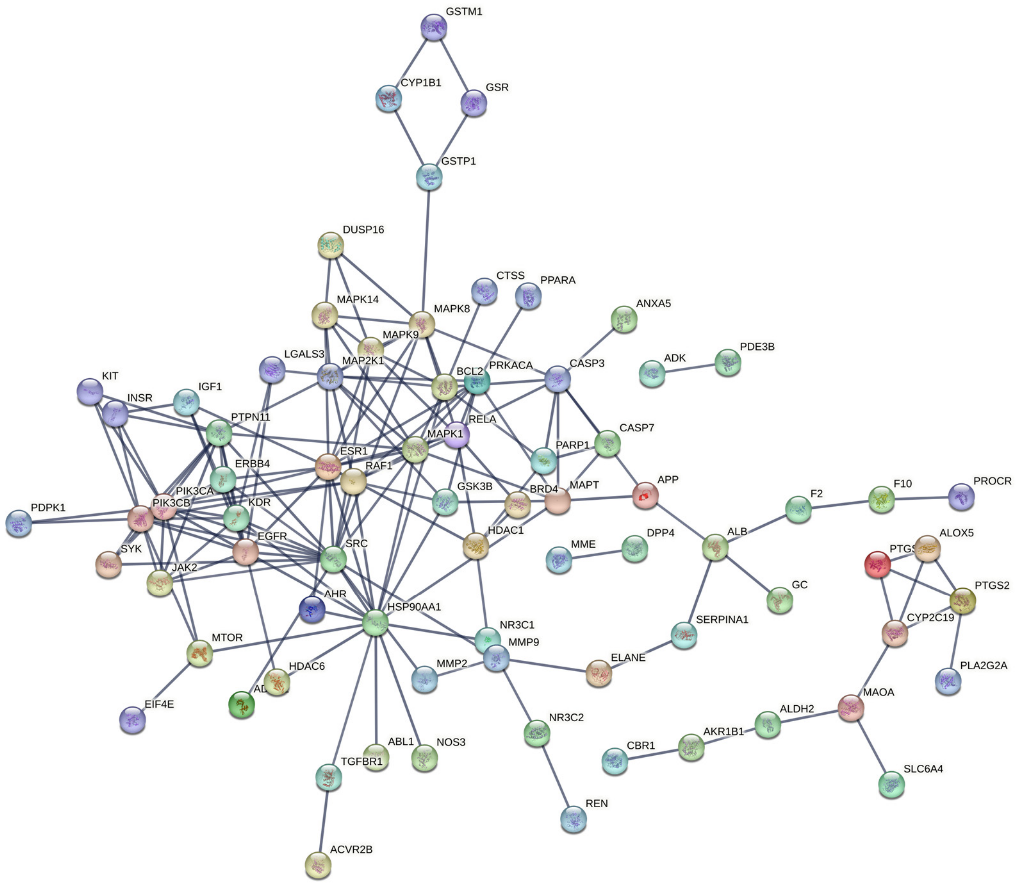

Figure S2. PPI network diagram of intersecting targets based on the STRING database.

Figure S3

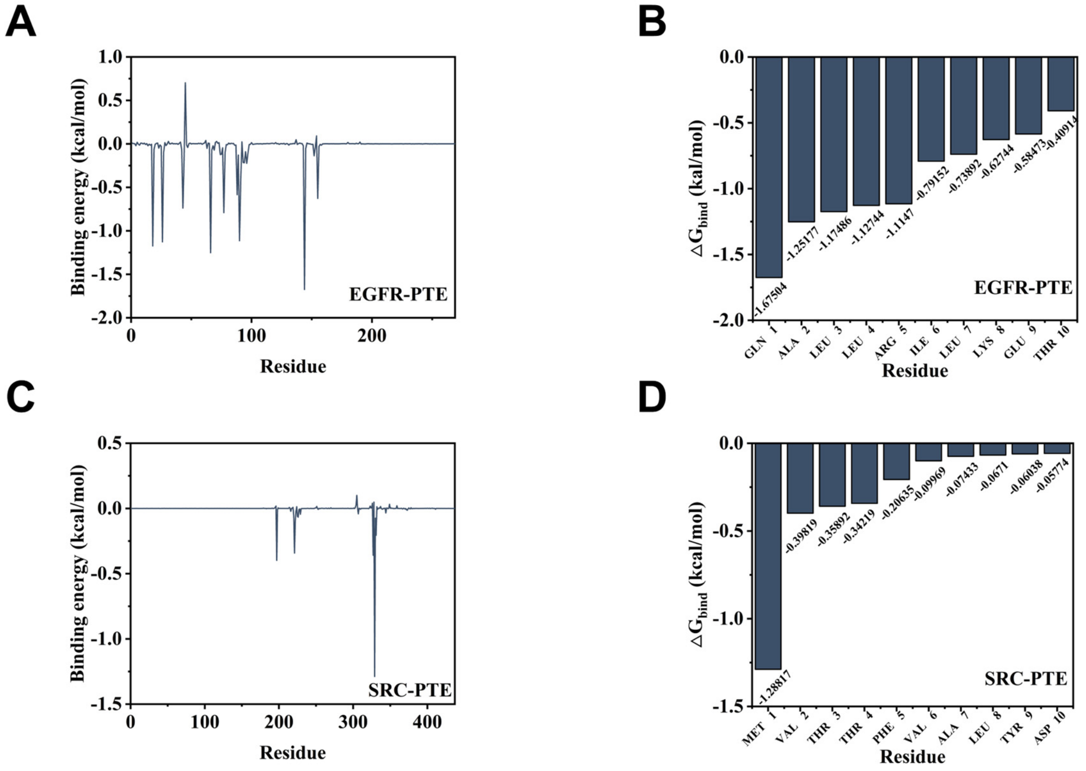

Figure S3. Contribution of AAs to the binding energy in target-PTE complex. (**A**) Binding energy contributions of the AAs in EGFR-PTE complex. (**B**) Top 10 AA residues contributing to binding energy in EGFR-PTE complex. (**C**) Binding energy contributions of the AAs in SRC-PTE complex. (**D**) Top 10 AA residues contributing to binding energy in SRC-PTE complex. AAs: amino acids.

Figure S4

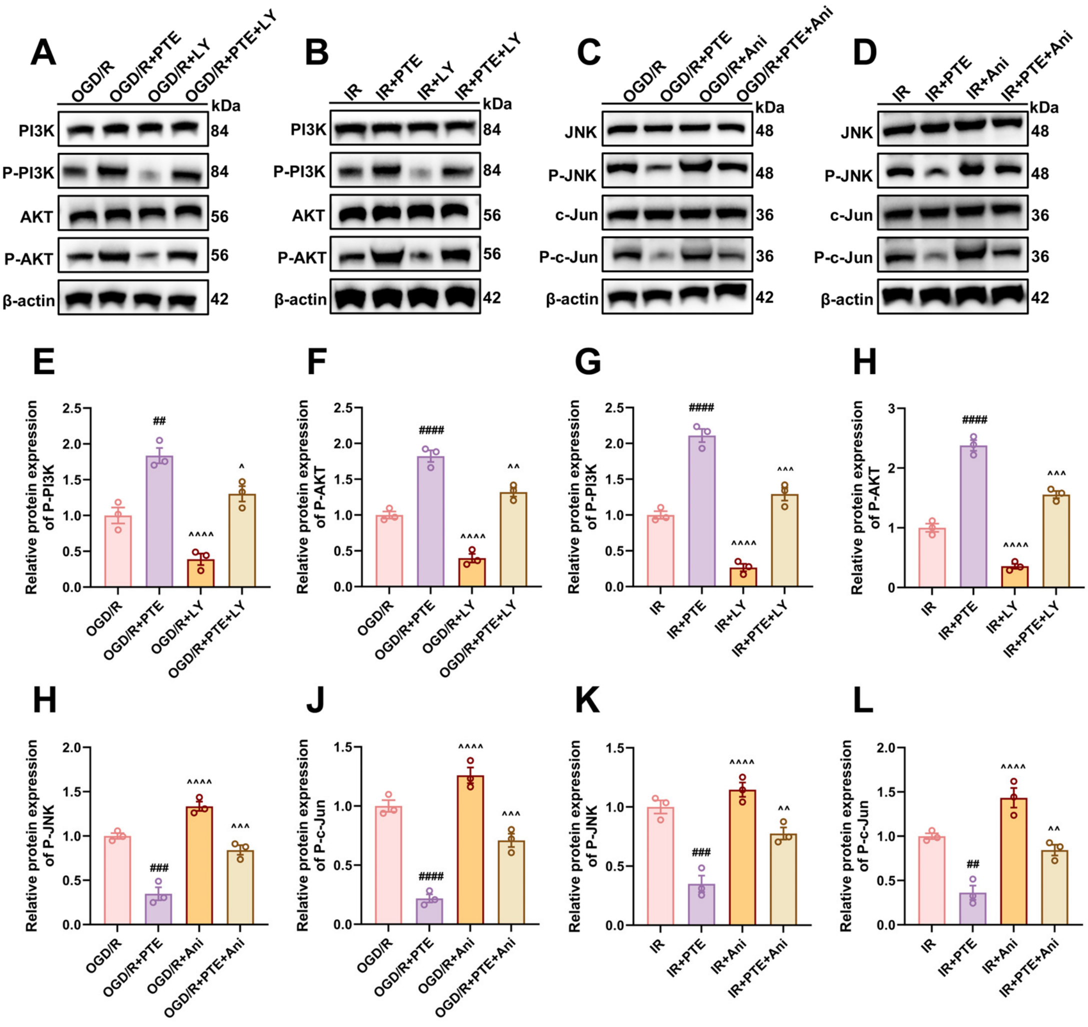

Figure S4. The impact of the PI3K inhibitor and JNK agonist on relevant signaling pathways. PTE was administered at a high dose (60 mg/kg, i.p. in vivo; 20 μM in vitro) at the start of reperfusion or reoxygenation. Both LY294002 (0.3 mg/kg via tail vein injection in vivo; 10 μM in vitro) and Anisomycin (15 mg/kg, i.p. in vivo; 20 μg/ml in vitro) were given 30 min prior to modeling. (**A, E-F**) *In vitro*, the impact of LY on protein expression in the PI3K/AKT pathway and its quantitative analysis. (**B, G-H**) *In vivo*, the impact of LY on protein expression in the PI3K/AKT pathway and its quantitative analysis. (**C, H-J**) *In vitro*, the impact of Ani on protein expression in the JNK/c-Jun pathway and its quantitative analysis. (**D, K-L**) *In vivo*, the impact of Ani on protein expression in the JNK/c-Jun pathway and its quantitative analysis. Data are presented as the mean ± SD (n = 3). $^{\#}P < 0.05$, $^{\#\#}P < 0.01$, $^{\#\#\#}P < 0.001$, $^{\#\#\#\#}P < 0.0001$, vs. IR group or OGD/R group; $^{\wedge}P < 0.05$, $^{\wedge\wedge}P < 0.01$, $^{\wedge\wedge\wedge}P < 0.001$, $^{\wedge\wedge\wedge\wedge}P < 0.0001$, vs. IR+PTE or OGD/R+PTE group; n.s. = non-significant. IR:

ischemia-reperfusion, OGD/R: oxygen-glucose deprivation/reoxygenation, LY: LY294002 (a PI3K inhibitor), Ani: Anisomycin (a JNK agonist), i.p.: intraperitoneal injection.

2. Supplementary Tables

Table S1

Table S1. The primer sequences used for RT-qPCR

Gene	Primer Sequences
Human-EGFR	F: GTGTGCCACCTGTGCCATCC<br>R: GCCACCACCAGCAGCAAGAG
Human-MAPK8	F: ACTACAGAGCACCCGAGGTCATC<br>R: TTTCTCCCATGATGCACCCAACTG
Human-PIK3CB	F: CTTTGCGACAAGACTGCCGAGAG<br>R: CGCCTGAAGCTGAGCAACATCC
Human-SRC	F: TGCGAGAAAGTGAGACCACGAAAG<br>R: ACTGGGTGCGGGAGGTGATG
Rat-EGFR	F: CACTACGCCGCCTGCTTCAAG<br>R: ACTGTGCCAAATGCTCCTGAACC
Rat-MAPK8	F: CCACCACCAAAGATCCCTGACAAG<br>R: GACGCCATTCTTAGTTCGCTCCTC
Rat-PIK3CB	F: GCTGGCTTGGACCTGCGAATG<br>R: GCGATTGTCTCAGAGGTGCTCAC
Rat-SRC	F: TCCCACATCCAAGCCTCAGACC<br>R: CATCCACACCTCTCCGAAGCAAC

Table S2

Table S2. Intersection targets of PTE and LIRI

Common Targets				
PTGS1	ESR1	PTGS2	SLC6A3	ADRB2
ABCB1	CA9	APP	PIK3CA	MAOA
HMGCR	ALOX5	MIF	ELANE	CHEK1
MAPK10	MAPK9	ACVR2B	LATS2	JAK2
ABL1	EPHX2	PIM1	ALB	PPIA
ADAM17	CASP7	MMP13	HSP90AA1	NR3C2
F2	DPP4	PDE5A	KDR	PLAU
TGFBR1	TPI1	NR3C1	SERPINA1	CBR1
MMP8	REN	MMP2	TEK	IL2
GLO1	CASP3	MAP2K1	LGALS3	VDR
RAC1	KIT	GSTM1	PROCR	RELA
SLC6A4	PRKACA	CYP1B1	AHR	SYK
PIK3CB	EGFR	MAPT	CYP2C19	MAPK14
HDAC1	BRD4	RAF1	MAPK8	MAPK1
PRF1	BCL2	HDAC6	MTOR	GC
NOS3	AKR1B1	ANXA5	F10	PDE3B
SRC	ALDH2	SOD2	PTPN11	NR1H2
NQO1	ERBB4	GSK3B	ADK	MME
PARP1	FABP3	IGF1	GSTP1	CTSS
MMP9	PDPK1	PLA2G2A	PPARA	SELE
INSR	CASP1	EIF4E	GSR	

Table S3

Table S3. STRING-based PPI network features of intersecting targets

Network characteristics	Value
Number of nodes	104
Number of edges	166
Average node degree	3.19
Local clustering coefficient	0.389
Expected number of edges	48
PPI enrichment p-value	$<1.0 \times 10^{-16}$

Table S4

Table S4. Top 10 hub genes in the network by CytoNCA

Target	Degree	Betweenness	Closeness	Clustering Coefficient
SRC	17	790.70508	34.79286	0.27941
HSP90AA1	16	1,100.65812	34.48333	0.11667
PIK3CA	14	205.53676	30.78452	0.35165
ESR1	14	446.25895	33.54286	0.25275
PIK3CB	13	132.48134	29.70119	0.37179
PTPN11	12	161.32722	30.02619	0.40909
EGFR	11	138.89271	30.74286	0.38182
MAPK1	11	338.10368	32.23333	0.32727
MAPK8	10	634.57505	30.9	0.28889
RAF1	9	135.11805	29.99286	0.36111

Table S5

Table S5. Top 10 hub genes in the network by Network Analyzer

Target	Degree	Betweenness Centrality	Closeness Centrality	Topological Coefficient
SRC	9	0.12314815	1.00000000	0.67901235
PTPN11	8	0.08750000	0.90000000	0.70833333
PIK3CA	8	0.05370370	0.90000000	0.73611111
EGFR	7	0.02731481	0.81818182	0.77777778
PIK3CB	7	0.01805556	0.81818182	0.77777778
ERBB4	6	0.01250000	0.75000000	0.81481481
JAK2	6	0.00555556	0.75000000	0.81481481
KDR	5	0.00000000	0.69230769	0.84444444
ESR1	5	0.02777778	0.69230769	0.73333333
MAPK1	3	0.00555556	0.60000000	0.81481481

Table S6

Table S6. Molecular docking results of Pterostilbene and core targets

Target	PDB ID	Docking site	Docking Score
EGFR	8A27	23.814833, −10.622104, −11.554250	−7.4
MAPK8	2XRW	11.837037, 6.743482, 19.451444	−7.2
PIK3CB	AF-P42338	−0.415906, −0.346282, −1.485532	−7.1
SRC	1FMK	−11.878750, 19.305125, 27.663062	−6.7
HSP90AA1	5J80	0.936686, 14.100467, −20.136760	−6.1
MAPK1	8AOJ	9.141650, 10.326875, 43.258300	−6.1
PIK3CA	9ASF	−73.994213, −0.626714, 26.363643	−5.9
ESR1	7BAA	17.457158, 19.994211, 0.223316	−5.9
PTPN11	3ZM1	20.878561, 33.313953, 26.392488	−5.8
RAF1	3IQU	−17.780500, −14.588100, 9.501800	−5.6

Table S7

Table S7 Binding energies and components predicted by MM/GBSA

Complex	Energy Component	Quercetin/PARP1 (kcal/mol)
PTE-EGFR	ΔE_{vdw}	−36.2928 ± 2.3984
	ΔE_{elec}	−10.3729 ± 2.4045
	ΔG_{GB}	26.0364 ± 4.0195
	ΔG_{SA}	−5.5367 ± 0.2840
	ΔG_{bind}	−26.1660 ± 2.8082
PTE-SRC	ΔE_{vdw}	−12.8409 ± 6.8596
	ΔE_{elec}	−2.5886 ± 3.7393
	ΔG_{GB}	9.4924 ± 5.6560
	ΔG_{SA}	−1.7313 ± 0.9975
	ΔG_{bind}	−7.6683 ± 4.7421

Abbreviations: ΔE_{vdw}: van der Waals energy, ΔE_{elec}: electrostatic energy, ΔG_{GB}: electrostatic contribution to solvation, ΔG_{SA}: non-polar contribution to solvation, ΔG_{bind}: binding free energy.

Table S8

Table S8. Top 10 amino acid residues involved in MD simulation

Molecules under simulation	Residue	Mean	SD
EGFR and Pterostilbene	GLN 1	−1.67504	0.00454
	ALA 2	−1.25177	0.00359
	LEU 3	−1.17486	0.0015
	LEU 4	−1.12744	0.00443
	ARG 5	−1.1147	0.00404
	ILE 6	−0.79152	0.00243
	LEU 7	−0.73892	0.00867
	LYS 8	−0.62744	0.0017
	GLU 9	−0.58473	0.0016
	THR 10	−0.40914	0.00137
SRC and Pterostilbene	MET 1	−1.28817	0.00037
	VAL 2	−0.39819	0.00000
	THR 3	−0.35892	0.00037
	THR 4	−0.34219	0.00037
	PHE 5	−0.20635	0.00000
	VAL 6	−0.09969	0.00037
	ALA 7	−0.07433	0.00050
	LEU 8	−0.0671	0.00050
	TYR 9	−0.06038	0.00069
	ASP 10	−0.05774	0.00037
